# Serum biomarkers associated with SARS-CoV-2 severity

**DOI:** 10.1038/s41598-022-20062-5

**Published:** 2022-09-26

**Authors:** Fabiani de Morais Batista, Marco Antonio Moreira Puga, Patricia Vieira da Silva, Roberto Oliveira, Paulo Cesar Pereira dos Santos, Bruna Oliveira da Silva, Mariana Bento Tatara, Daniel Henrique Tsuha, Maria Aparecida dos Santos Pires, Crhistinne Cavalheiro Maymone Gonçalves, Rômulo Pessoa e Silva, Nathália Tavares Ferreira, Amanda Pinheiro de Barros Albuquerque, Giselle da Silva Duarte, Márcia Edilaine Lopes Consolaro, Fabio Juliano Negrão, Idalina Cristina Ferrari, Luciano Pamplona de Goes Cavalcanti, Karen Soares Trinta, Guilherme S. Ribeiro, Moacyr Jesus Barreto de Melo Rêgo, Rosemary J. Boyton, André Machado Siqueira, Daniel M. Altmann, Julio Croda

**Affiliations:** 1 School of Medicine, Federal University of Mato Grosso do Sul, Campo Grande, Brazil; 2 Associated Prof. Nursing Course., State University of Mato Grosso do Sul, Dourados, MS Brazil; 3 Laboratory of Research in Health Science, Faculty of Health Science, Federal University of Grande Dourados, Dourados, Brazil; 4 State Health Department of Mato Grosso do Sul, Center for Strategic Information in Health Surveillance, Campo Grande, Brazil; 5 Faculty of Health Sciences, Federal University of Grande Dourados, Dourados, Mato Grosso do Sul Brazil; 6 Universitary Hospital of Federal University of Grande Dourados, Federal University of Grande Dourados, Dourados, Brazil; 7 Federal University of Pernambuco – UFPE, Recife, Brazil; 8 Laboratory in Clinical Research of Acute Febrile Illnesses, National Institute of Infectious Diseases Evandro Chagas, Oswaldo Cruz Foundation, Rio de Janeiro, Brazil; 9 Postgraduate Program in Biosciences and Pathophysiology, State University of Maringá, Maringá, PR Brazil; 10 Regional University Hospital of Maringá, State University of Maringá, Maringá, Brazil; 11 Postgraduate Program in Public Health, Federal University of Ceará, Fortaleza, Brazil; 12 School of Medicine, Christus University Center, Fortaleza, Brazil; 13 Postgraduate Program in Pathology, Federal University of Ceará, Fortaleza, Brazil; 14 Laboratory of Diagnostic Technology, Bio-Manguinhos, Fiocruz, Rio de Janeiro, Brazil; 15 Instituto Gonçalo Moniz, Fundação Oswaldo Cruz, Salvador, Brazil; 16 School of Medicine, Federal University of Bahia, Salvador, Brazil; 17 Department of Infectious Disease, Imperial College London, London, UK; 18 Lung Division, Royal Brompton and Harefield Hospitals, London, UK; 19 National Institute of Infectious Diseases Evandro Chagas, Oswaldo Cruz Foundation, Rio de Janeiro, Brazil; 20 Department of Immunology and Inflammation, Imperial College London, London, UK; 21 Fiocruz Mato Grosso do Sul, Oswaldo Cruz Foundation, Campo Grande, Brazil

**Keywords:** Viral infection, Infectious diseases

## Abstract

Immunity with SARS-CoV-2 infection during the acute phase is not sufficiently well understood to differentiate mild from severe cases and identify prognostic markers. We evaluated the immune response profile using a total of 71 biomarkers in sera from patients with SARS-CoV-2 infection, confirmed by RT-PCR and controls. We correlated biological marker levels with negative control (C) asymptomatic (A), nonhospitalized (mild cases-M), and hospitalized (severe cases-S) groups. Among angiogenesis markers, we identified biomarkers that were more frequently elevated in severe cases when compared to the other groups (C, A, and M). Among cardiovascular diseases, there were biomarkers with differences between the groups, with D-dimer, GDF-15, and sICAM-1 higher in the S group. The levels of the biomarkers Myoglobin and P-Selectin were lower among patients in group M compared to those in groups S and A. Important differences in cytokines and chemokines according to the clinical course were identified. Severe cases presented altered levels when compared to group C. This study helps to characterize biological markers related to angiogenesis, growth factors, heart disease, and cytokine/chemokine production in individuals infected with SARS-CoV-2, offering prognostic signatures and a basis for understanding the biological factors in disease severity.

## Introduction

COVID-19 has a broad spectrum of clinical manifestations. Studies estimate that 30 to 60% of COVID-19 cases are asymptomatic or mildly symptomatic, and 5% of symptomatic cases are seriously ill^1^, which is a reflection of complex interactions, including immunological, inflammatory, and coagulative cascades^2^.

Differences in host responses to severe acute respiratory syndrome coronavirus 2 (SARS-CoV-2) play a decisive role in COVID-19 with respect to differential clearance of viral infection but also have the potential for immunopathological damage to the host^2^. Immunological dysregulation and an increase in the level of cytokines (proinflammatory, profibrotic, and regulatory of the immune response) are responsible for tissue damage^3–5^.

Vascular alterations are other characteristics associated with COVID-19^5,6^ with changes in the extremities, suggesting thrombotic microangiopathy^7^. Coagulation-related factors have also been associated with multisystem organ failures, such as diffuse intravascular coagulation and large-vessel thrombosis^8,9^.

The severity of COVID-19 appears to be related to an exacerbated immune response and events associated with vascular injury^1,3,10,11^ Several studies have documented the association between COVID-19 severity and circulating levels of C-reactive protein (CRP), interleukin-6, and D-dimer^12–16^. A better understanding of the broad spectrum of immune responses that differentiate mild from severe cases is critical for identifying future biomarkers and developing new, specific therapies for COVID-19. Also, it is important to understand if the variation in immunological response against the infection differs among populations with distinct genetic profile. In this the first study in Brazil, we evaluated three panels (Angiogenesis and growth factor, Cardiovascular Diseases, Cytokines/Chemokines) giving a total of 71 biomarkers in sera from asymptomatic, mild and severe COVID-19 patients.

## Results

From individuals eligible according to the selection criteria, we selected 48 participants using non-probability sampling, for convenience, divided into four groups: 9 Negative Control (C), 14 Asymptomatic (A), 13 Non-hospitalized (Mild Cases-M), and 12 Hospitalized (Severe cases-S) (of these, five were admitted to the Intensive Care Unit and one died).

The majority were female (67%), had a median age of 47 years and 50% to the black ethnic group. (Table 1). The most common signs and symptoms were headache, fever, runny nose, cough, anosmia, and dysgeusia (Table 2).

**Table 1:** Sociodemographic characteristics according to clinical outcome in individuals with SARS-CoV-2 infection and negative controls (n = 48).

Variables	Controls-C N = 9	Asymptomatic-A N = 14	Mild Cases-M N = 13	Severe Cases-S N = 12	*p*-value
**Gender**					
Female	4 (44.4%)	10 (71.4%)	11 (84.6%)	7 (58.3%)	0.234^**a**^
Male	5 (55.6%)	4 (28.6%)	2 (15.4%)	5 (41.7%)	
**Age y**					
Median (IQR)	43 (32.0–49.0)	49.5 (21.50–54.25)	40 (33.0–44.0)	62.5 (58.5–66.5)	0.005^**b**^
**Ethnicity**					
White	1 (11.1%)	6 (42.9%)	4 (30.8%)	10 (83.3%)	0.010^**a**^
Black	8 (88.9%)	7 (50.0%)	7 (53.8%)	2 (16.7%)	
Others	0 (0.0%)	1 (7.1%)	2 (15.4%)	0 (0.0%)	

*y* years: *IQR* Interquartile Range: *Mild Cases-M*: Non-hospitalized; *Severe cases-S*: Hospitalized.

^**a**^ Fisher’s exact test.

^**b**^ Kruskal–Wallis test.

**Table 2:** Signs and symptoms according to clinical outcome in individuals with SARS-CoV-2 infection and negative controls (n = 48).

Variables	Controls-C N = 9	Asymptomatic-A N = 14	Mild cases-M N = 13	Severe cases-S N = 12	*p*-value
**Fever**					
Yes	0 (0.0%)	0 (0.0%)	8 (61.5%)	7 (58.3%)	< 0.001^**a**^
Median (IQR)*	0 (0–0)	0 (0–0)	3 (0–4.0)	3.5 (0–5.5)	< 0.001^**b**^
**Cough**					
Yes	0 (0.0%)	0 (0.0%)	7 (53.8%)	9 (75.0%)	< 0.001^**a**^
Median (IQR)*	0 (0–0)	0 (0–0)	3 (0–6.0)	3.5 (2.25–5.0)	< 0.001^**b**^
**Sore throat**					
Yes	0 (0.0%)	0 (0.0%)	2 (15.4%)	5 (41.7%)	0.009^**a**^
Median (IQR)*	0 (0–0)	0 (0–0)	0 (0–0)	0 (0–5.0)	0.014^**b**^
**Nasal congestion**					
Yes	0 (0.0%)	0 (0.0%)	9 (69.2%)	7 (58.3%)	< 0.001^**a**^
Median (IQR)*	0 (0–0)	0 (0–0)	4 (0–7.0)	3.5 (0–5.0)	< 0.001^**b**^
**Dyspnea**					
Yes	0 (0.0%)	0 (0.0%)	1 (7.7%)	9 (75.0%)	< 0.001^**a**^
Median (IQR)*	0 (0–0)	0 (0–0)	0 (0–0)	4 (1.5–5.25)	< 0.001^**b**^
**Chest pain**					
Yes	0 (0.0%)	0 (0.0%)	1 (7.7%)	1 (8.3%)	0.690^**a**^
Median (IQR)*	0 (0–0)	0 (0–0)	0 (0–0)	0 (1.5–5.25)	0.598^**b**^
**Headache**					
Yes	0 (0.0%)	0 (0.0%)	9 (69.2%)	4 (33.3%)	< 0.001^**a**^
Median (IQR)*	0 (0–0)	0 (0–0)	4 (0–7.0)	0 (0–1.75)	< 0.001^**b**^
**Loss of smell**					
Yes	0 (0.0%)	0 (0.0%)	8 (61.5%)	3 (25.0%)	< 0.001^**a**^
Median (IQR)*	0 (0–0)	0 (0–0)	2 (0–3.0)	0 (0–0.25)	0.003^**b**^
**Loss of taste**					
Yes	0 (0.0%)	0 (0.0%)	8 (61.5%)	3 (25.0%)	< 0.001^**a**^
Median (IQR)*	0 (0–0)	0 (0–0)	2 (0–4.0)	0 (0–0.25)	0.003^**b**^

*Mild Cases-M* Non-hospitalized; *Severe Cases-S* Hospitalized.

^a^ Fisher’s exact test.

^b^ Kruskal–Wallis test.

*Median of days between symptom onset and recruitment date (IQR, interquartile range).

We sequenced 12/48 samples, and the variants were identified as nine Zeta (P2), 2 Gamma Subvariant (P.1.7 and 1 N.9) and one B.1.1.

### Cytokines/chemokines

**Figure 1:**
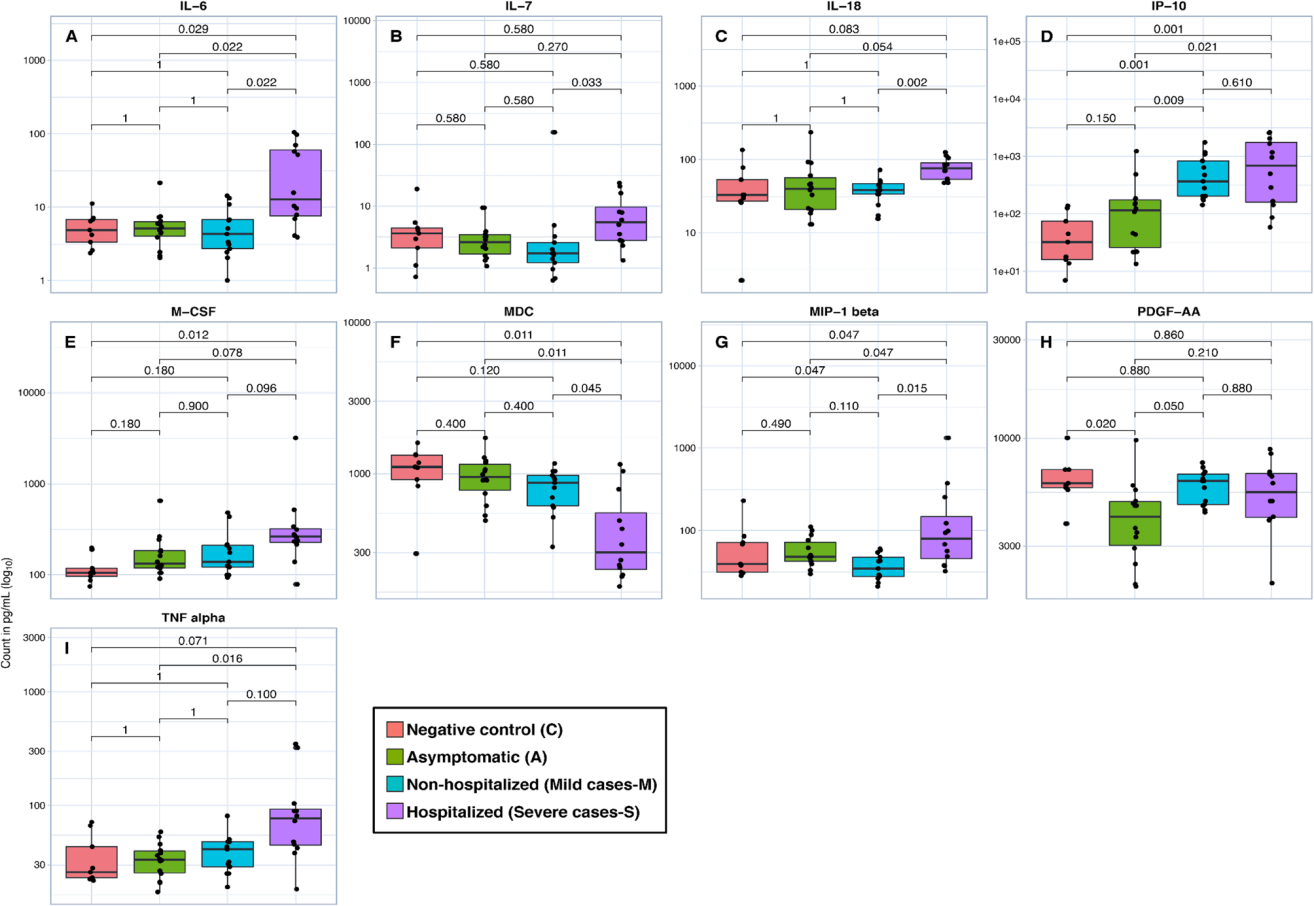
Box plot representation of the serum concentration (pg/mL) of cytokines/chemokines. Levels of IL-6 (**A**), IL-7 (**B**), IL-18 (**E**), IP-10 (**G**), M-CSF (**H**), MDC (**I**), MIP-1 beta (**K**), PDGF-AA (**L**), and TNF alpha (**M**). The top and bottom lines of boxes are the 25th and 75th percentiles, respectively, and the band in the middle of the box is the median. analysis. Statistical analysis was performed using the Mann–Whitney U test between the negative control (C), asymptomatic (A), nonhospitalized (mild cases-M), and hospitalized (severe cases-S) groups.

Of the 44 cytokines/chemokines evaluated, 9 showed a statistically significant difference between the groups (IL-6, IL-7, IL-18, IP-10, M-CSF, MDC, MIP-1 beta, PDGF-AA and TNF alpha). This study identified important differences in cytokines and chemokines according to clinical evaluation: IL-6, IP-10, M-CSF, MDC and MIP-1 beta were higher in severe cases than in the C group. MDC biomarkers from hospitalized participants presented at a lower level compared to the control group. When comparing C with A, there were lower levels of PDGF-AA in the A group. The M group had lower levels of MDC and higher levels of IP-10 than the C group (Fig. 1).

### Cardiovascular disease markers

**Figure 2:**
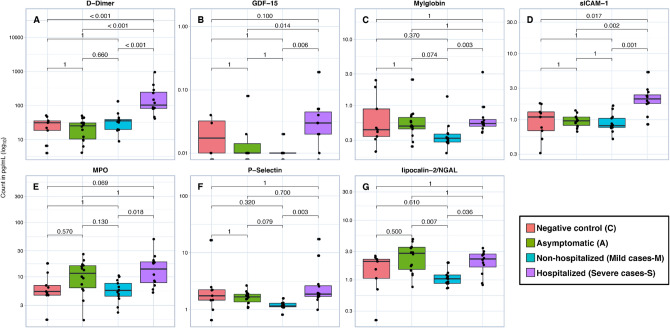
Box plot representation of serum concentration (pg/mL) of cardiovascular biomarkers. Levels of D-Dimer (**A**), GDF-15 (**B**), Mylglobin (**C**), sICAM-1 (**D**), MPO (**E**), P-Selectin (**F**), and Lipocalin-2/NGAL (**G**). The top and bottom lines of boxes are the 25th and 75th percentiles, respectively, and the band in the middle of the box is the median. Statistical analysis was performed using the Mann–Whitney U test between the negative control (C), asymptomatic (A), nonhospitalized (mild cases-M), and hospitalized (severe cases-S) groups.

Among the 10 cardiovascular disease markers tested, 7 showed significant differences between groups (D-dimer, GDF-15, myoglobin, sICAM-1, MPO, P-selectin and lipocalin-2/NGAL). This study identified differences in biomarkers according to the clinical disease outcome: D-dimer, and sICAM-1 biomarkers from severe case group participants were the highest. Myoglobin and P-selectin biomarkers from M were the lowest compared with S and A. There was no difference between biomarkers from C and A (Fig. 2).

### Angiogenesis and growth factor markers

**Figure 3:**
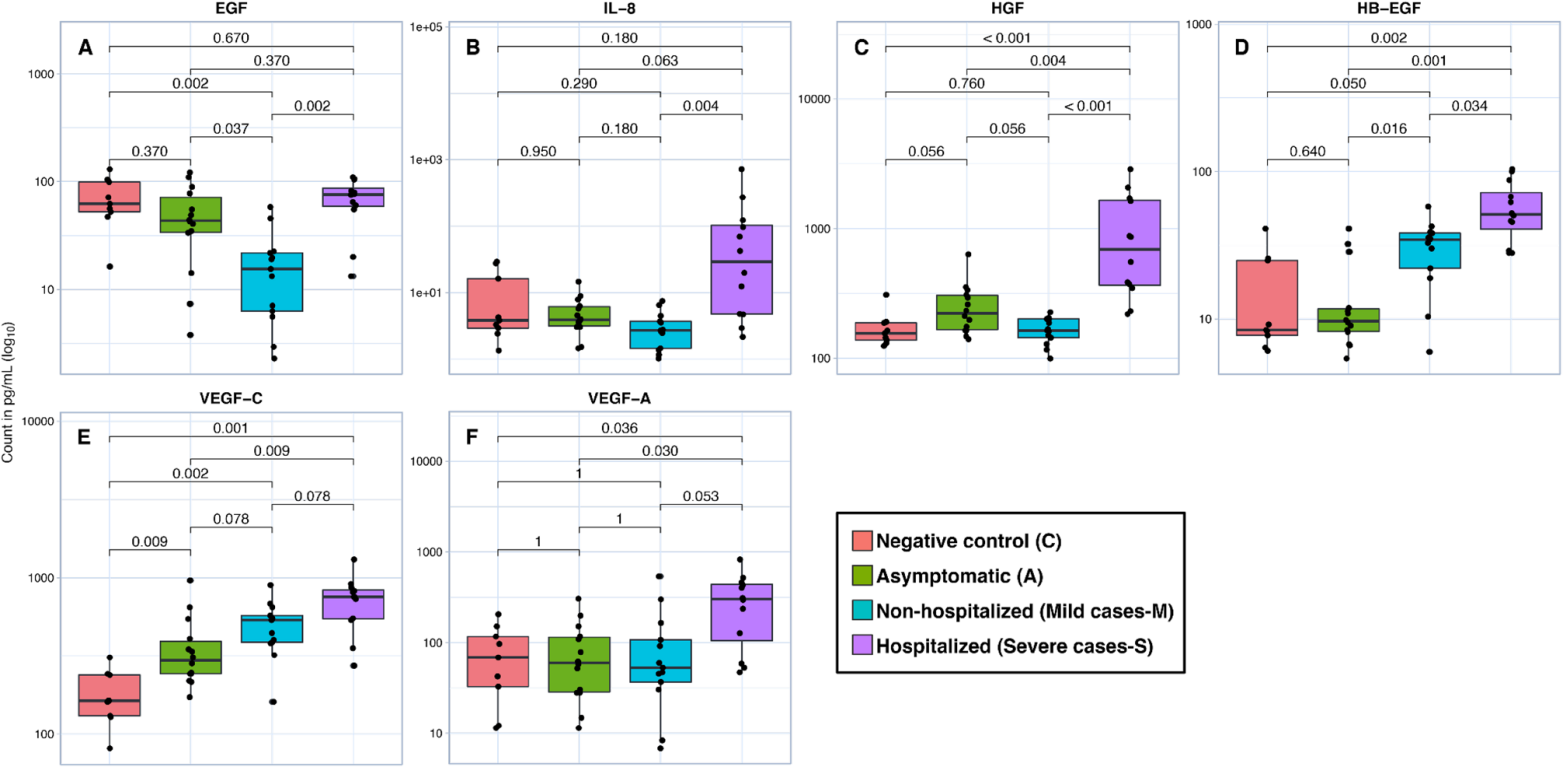
Box plot representation of the serum concentration (pg/mL) of angiogenesis biomarkers. Levels of EGF (**A**), IL-8 (**B**), HGF (**C**), HB-EGF (**D**), VEGF-C (**E**), and VEGF-A (**F**). The top and bottom lines of the boxes are the 25th and 75th percentiles, respectively, and the band in the middle of the box is the median. Statistical analysis was performed using the Mann–Whitney U test between the negative control (C), asymptomatic (A), nonhospitalized (mild cases-M), and hospitalized (severe cases-S) groups.

Among the 17 angiogenesis markers evaluated, we identified 6 that showed significantly different levels between groups (EGF, IL-8, HGF, HB-EGF, VEGF-C, VEGF-A), always with higher levels of these biomarkers in the most severe group that progressed to hospitalization compared to the others (C, A and M). The EGF biomarker from mild cases was the lowest compared to the S, A, and C groups (*p* < 0.05). Comparing A to C, there was a significant difference for VEGF-C (*p* < 0.05). The VEGF-C biomarker from C was the lowest (Fig. 3).

## Discussion

Of the 71 serum biomarkers analyzed here, 22 (EGF, IL-8, HGF, HB-EGF, VEGF-C, VEGF-A, D-dimer, GDF-15, Myoglobin, sICAM-1, MPO, P-Selectin, Lipocalin-2/NGAL, IL-6, IL-7, IL-18, IP-10, M-CSF, MDC, MIP-1 beta, PDGF-AA and TNF alpha) showed significant differences in their levels when comparing patients with SARS-CoV-2 infection to controls. We found that most of these biomarker alterations were present in severe cases. The S group was skewed to the elderly, with a median age of 62.5 (*p* = 0.005), and for the white ethnicity group with 83.3%. The most common symptoms were cough (75.0%; *p* < 0.001), sore throat (41.7%; *p* = 0.014) and dyspnea (75.0%; *p* < 0.001). These findings suggest that dysregulation of the immune response, especially in the elderly population, contributes to worsening of the disease in the acute phase.

The occurrence of the intense inflammatory process, generated in response to infection by SARS-CoV-2, damages the tissues, in which the patient may have respiratory distress, kidney failure, or heart problems, causing serious complications, with a worsening of the clinical condition, that may progress to death^1,15,17^. In this study, most participants in the severe cases group showed important changes in the biomarkers that are part of the inflammatory process, making it possible to analyze them together with the clinical course of COVID-19 and the severity of the disease.

Cytokines, proteins that modulate the function of other cells or the cells they are generated, play important roles during COVID-19 and are related to the severity of viral infection when hyperstimulated^18,19^. A inflammatory network associated with the severity of COVID-19 is formed by cytokines (IP-10, IL-6, IL-7 and VEGF-α), that are in significantly elevated concentrations in cases of severe pneumonia compared to mild symptoms^20^.

In this study, it was possible to verify that the EGF was elevated in severe cases when compared to the M group (*p* = 0.002). Considering that EGF is a ligand of the epidermal growth factor receptor (EGFR), it initiates an intracellular signaling cascade by linking the extracellular domains of these receptors. Therefore, this signaling increases the potential for cell proliferation, angiogenesis, and resistance to apoptosis^2^. Other ligands for this receptor are heparin-binding growth factor (HB-EGF) and amphiregulin^21–23^. HB-EGF is among the markers of angiogenesis and growth factor; therefore, it is inferred that the change in EGF is closely linked to the change in angiogenesis as part of the process that leads to the severity of the infection.

The action of the immune response causes an acute inflammatory reaction by the action of macrophages, which secrete cytokines, such as interleukin-6 (IL-6) and induced protein 10 (IP-10)^24^. Both the proinflammatory cytokines IL-6 and IP-10 are activators of interferon-gamma (IFN-γ), and IL-27 and IL-17 production^25^. IL-6 stimulates acute phase protein synthesis, neutrophil production and B lymphocyte growth and inhibits the generation of regulatory T cells. IP-10 also regulates innate and adaptive immune responses, being an important signaler, through the increase in calcium inside the cell, and the more this happens, it increases phagocytic action, liberating more cytokines^26^. High levels of IL-6, and IP-10 were found in severe cases when compared to the negative control group (*p* = 0.029, 0.001, respectively). This result suggests the presence of signals for an excessive inflammatory process in patients with COVID-19 and more commonly in critically ill patients than in nonsevere patients. A higher concentrations of IL-6 were associated with ward and ICU patients, it might contribute to use alternative therapeutic intervention with anti-IL6 in the clinical course^27^.

The proinflammatory cytokines IL6, IL8 and TNF-alpha may be related to the D-dimer elevation. This study found high levels of IL-6, IL-8 and TNF-alpha in the severe cases when compared to the M, A and C groups. Furthermore, we identified higher levels of cytokines with direct roles in the adaptive immune response (IL-7, and IL-18) among severe cases and M groups. IL-7 is essential for the survival of mature and memory T cells, and and IL-18 promote and proliferate T lymphocytes, respectively^25,28^. IL-6 is an important marker of disease severity and is a mortality predictor. However, IL-6 also plays an important role in monitoring the therapeutic response^29^. Another study also showed that IL-6 levels are higher in patients with sepsis than in COVID-19 cases; however, IL-6 levels are still elevated in severe cases of COVID-19 compared to healthy patients^30^. Despite this, it is not possible to confirm if the IL-6 was elevated due to the severity of the disease. The severe group has a higher median age (*p* = 0.005), that can contribute to elavated IL-6^31,32^.

MDC cytokines were reduced in hospitalized COVID-19 patients in this study, which was observed by Ling et al. (2021). This chemokine is responsible for maintaining the homeostasis of the mucosal barrier and has a protective function in inflammatory diseases, attracting macrophages to phagocytosis. Therefore, we can suggest that there was a decrease in the presence of macrophages or that only their functionality could have been inhibited, causing a breakdown in the homeostasis of the epithelium and leading to an inflammatory profile. Ling et al. (2021) suggested that the biomarker with the best performance in the late phase (8–12 days after the onset of the disease) to predict severity is the MDC^33^**.**

This study found that heart disease markers such as D-dimer, and sICAM-1 were elevated in severe cases. Measuring D-dimer levels has been used to predict disease severity. D-dimer has been widely used as a biomarker for thrombotic disorders, once as a fibrin degradation product^19,34^. D-dimer levels were higher when comparing the severe cases versus mild cases, asymptomatic and the negative control groups (*p* < 0.001). Some studies describe D-dimer as a predictive biomarker of mortality, which can be an easy-to-perform and low-cost laboratory indicator for prognosis and pretreatment to avoid thrombosis episodes^35,36^.

Although the levels of GDF-15 were lower in the asymptomatic and non-hospitalized (mild) cases compared to the control group, these differences were not statistically significant (*p* = 1.00). Thus, it is not possible to ascertain whether there is a downregulation of the GDF-15 production among subjects with asymptomatic or mild SARS-CoV-2 infection compared to subjects in the control group. In contrast, the GDF-15 levels were statistically greater among the subjects with severe COVID-19 compared to the asymptomatic and mild groups, but not in the comparison with the control group. The non-significant higher levels of GDF-15 among subjects with severe COVID-19 compared to controls may be due to insufficient power, given that increased levels of GDF-15 have been associated with an increased risk of ICU admission and mortality among COVID-19^37,38^, and with postponing withdrawal of mechanical ventilation and late recovery (Ebihara et al. 2021)^30^. Our findings, together with those from other studies suggest that GDF-15 is increased in patients with severe COVID-19, but not in asymptomatic or mild infections.

Soluble intercellular adhesion molecule 1 (sICAM-1) represents a circulating form of ICAM-1. The interaction between ICAM-1, present in endothelial cells, and LFA-1 facilitates the adhesion and migration of leukocytes through the endothelium. ICAM-1 and its circulating form have been implicated in the development of numerous diseases^39^. In this study, we found that this molecule presented high levels in group S compared to groups C, A and M and obtained significant differences, suggesting that sICAM-1 is involved in the inflammatory process of severe cases of SARS-CoV-2 infection. According to our study, group S presented high levels of TNF-alpha, which contributes to endothelial activation processes and vascular occlusion, suggesting that sICAM-1 is a predictor for severe cases of COVID-19.

Vascular endothelial growth factor (VEGF-A) presented significantly higher levels in the samples of the severe cases (S) than in the samples of the asymptomatic cases (A) and negative controls (C) (; *p* = 0.030 and *p* = 0.036, respectively). Yin et al.^40^ suggested that VEGF may be a notable target for the treatment of patients with SARS-CoV-2. This growth factor is related to increased vascular permeability at the pulmonary level, causing plasma extravasation and, later, pulmonary edema^41^. Anti-VEGF therapy has also been investigated for patients with neurological signs, since VEGF is related to the stimulation of an inflammatory response in the central nervous system (CNS) through lymphocyte recruitment and increased release of proinflammatory cytokines such as IL-6 and TNF-α^42^.

Vascular endothelial growth factor type C (VEGF-C) presented a different profile from that observed for VEGF-A. VEGF-C showed differences in concentrations between all groups when compared with the Negative Control (C). The levels in C were lower than those in A (*p* = 0.009), M (*p* = 0.002) and S (*p* = 0.001)^40,41^. The data suggest that the concentration of this glycoprotein increases with the severity of the disease, as they are related to the lymphatic system and endothelial cells, and the high levels cause increased vascular permeability and worsening of endothelial damage. Studies addressing VEGF-C in the context of COVID-19 are still quite uncommon, which reinforces the importance of deepening the investigation of the role of this biomarker in the immunopathology of COVID-19.

Similar to VEGF-A, hepatocyte growth factor (HGF) presented significantly higher levels in group S than in the other groups (M, A, and C), with even more pronounced differences (*p* < 0.001). Other studies have shown that the concentrations of this cytokine are higher in symptomatic patients or those with more severe clinical conditions than in groups of asymptomatic patients or those with fewer cases of COVID-19^24,43^. According to them, HGF elevation is a counterregulatory immune mechanism in response to the elevation of CXCL13 and other proinflammatory cytokines during SARS-CoV-2 infection^44^. In addition, Guo et al.^45^ identified a positive and significant correlation between high levels of HGF and IL-1β and the severity of COVID-19 determined by the APACHE II (Acute Physiology and Chronic Health Evaluation) score based on the dosage of these analytes in the peripheral blood of nine patients.

The evaluation of heparin-binding EGF-like growth factor (HB-EGF) showed an interesting profile in the study groups: although a significant difference in protein levels was obtained between groups S and M (with S presenting higher values), HB-EGF levels in the C and A groups were much lower compared to the two groups with symptomatic patients. The largest difference was between groups S and A (*p* = 0.001). There were no significant differences between the C and A groups. The data then suggest that it is a macromolecule that may be related to the development of symptoms in COVID-19 or that its elevation is due to some counterregulatory mechanism and that, like HGF, it can be used as a biomarker of severity. This protein has biological functions in several tissues, and among its activities are endometrial maturation and the protection of endometrial stromal cells^46,47^. The soluble form (sHB-EGF) is a potent mitogen and chemoattractive to a wide variety of cells, including vascular smooth muscle cells, fibroblasts and keratinocytes. HB-EGF has been implicated in different pathological processes, such as cardiac hypertrophy^48,49^, smooth muscle cell hyperplasia and atherosclerotic plaque formation^50–52^, oncogenic transformation^53,54^ and even hypertension and pulmonary fibrosis^55–57^.

There are some limitations to this study. The number of participants were similar among black (n = 24) and white (n = 21) ethnicity. However, there is differences in severe group (83.3% were white; *p* = 0.010). This results differ from previous studies which black ethnicity had higher risk for infection and hospitalization^58^ and probably related to convenience selection using no-probability sampling. Biomarker analysis was performed in the blood sample collected at patient enrollment, and no additional samples were obtained during the follow-up of the participants for comparative analysis. Another important limitation is that we do not have complete data on disease severity in patients who were hospitalized (i.e., use of mechanical ventilation, vasoactive drugs, dialysis, length of stay in an intensive care unit, and other aspects related to severity). The multiple comparisons may have led to the identification of casual differences, which do not necessarily represent reality, but rather a type II error related to sample variability, which may reflect in the lower levels of EGF in the mild cases than in the control group. Although this study analyzed a large number of biomarkers (71biomarkers) and suggested a correlation of some with the severity of COVID-19, we did not analyze other markers associated with the severity of COVID, such as CHI3L1 and IGFALS^59^, CXCL8, CXCL10, and CCL20^60^. Finally, this study is underpower and is not intended to be definitive or conclusive on the subject, but to contribute to the investigation of the relationship between biomarkers and disease severity.

Due to the rapid spread of COVID-19 and the high capacity of mutation of the SARS-COV-2 virus, it is necessary to better understand the biomarkers that describe the immune response associated with infection and how they correlate with disease severity. This is the first compressive study in Brazil that identified and described the immune response and biological markers related to angiogenesis and infection factors, heart disease, and cytokine production in SARS-CoV-2-infected individuals.

## Materials and methods

### Study population, eligibility criteria and data collection

This study is part of the Multicentric study of the Natural History of the Novel Coronavirus SARS-CoV-2 in Brazil (REBRACOVID). We included participants from four Brazilian cities (Campo Grande-MS, Dourados-MS, Maringá-PR and Recife-PE) with laboratory and analytical approaches from October 2020 to June 2021. Individuals over 18 years of age with clinical suspicion of flu-like symptoms (defined by the presence of flu-like symptoms for up to 11 days) and confirmed COVID-19 by quantitative reverse transcription polymerase chain reaction (RT-qPCR) were included (index cases). We collected swabs and blood samples from index cases and household contacts of index cases, regardless of whether the contacts had symptoms. The index cases and contacts were categorized according to RT-qPCR results and outcomes into four (4) groups: negative control (C), asymptomatic (A), nonhospitalized (mild cases-M), and hospitalized (severe cases-S).

For this study, we analyzed the data for participants who met the following criteria: (1) had RT-qPCR results; (2) had sufficient blood samples for all tests; (3) had complete data on symptoms and sociodemographic characteristics, and (4) fit into one of the four study groups (C, A, M and S).

### Biological samples and laboratory procedures

Nasopharyngeal swabs were collected for the detection of infection by SARS-CoV-2 by RT-qPCR using a mix of GoTaq Probe 1-Step RT-qPCR Systems, Promega, and specific primers and probes (IDT). Biomarker analysis was performed on serum samples. Aliquots with 500 μL of serum, stored in a − 20 °C freezer, were obtained from 15 mL of blood collected in Gel SST tubes and centrifuged at 2,000 rpm. Multiplex bead-based assays were performed using MAGPIX Luminex^®^ xMAP^®^ technology on serum samples from these participants using three different MILLIPLEX^®^ Map panels: (1) Human Cytokine/Chemokine/Growth Factor Panel A Magnetic Bead Panel, (2) Human Cardiovascular Disease (CVD) Magnetic Bead Panel 2 and (3) Human Angiogenesis/Growth Factor Magnetic Bead Panel 1. A total of 74 distinct bead populations were evaluated, these being analytes related to cytokines/chemokines (47), cardiovascular diseases (10), and angiogenesis and growth factor (17) (Supplementary Table 1).

### Data analysis

The values obtained through the Multiplex test were analyzed in R version 4.2.1 and RStudio 2022.02.2 software. The Kruskal–Wallis test was applied, and markers that presented *p* < 0.05 were selected and later analyzed through post hoc analysis by the Mann–Whitney U test and Bonferroni correction for multiple comparisons between groups. We used simple descriptive statistics to characterize the participants according to sex, age group, ethnicity, signs and symptoms.

### Ethics statements

This study was carried out with the approval of the Research Ethics Committee of the Federal University of Mato Grosso do Sul, under protocol number 4,383.724 CAAE 32874720.8.2001.0021. All research was performed in accordance with relevant guidelines and regulations. The Informed Consent Term was obtained from all participants.

## Supplementary Information

Supplementary Information.

## Data Availability

The raw data supporting the conclusions of this article will be made available by the authors, without undue reservation. To request the data can be made directly with the corresponding author Dr. Julio Croda.

## References

[1] BRASIL. Ministério da saúde. Diretrizes para diagnóstico e tratamento da Covid-19. Brasília-DF (2020).

[2] Samprathi M, Jayashree M (2020). Biomarkers in COVID-19: An up-to-date review. Front. Pediatr..

[3] Wang C (2020). Alveolar macrophage dysfunction and cytokine storm in the pathogenesis of two severe COVID-19 patients. EBioMedicine.

[4] Tay MZ, Poh CM, Rénia L, MacAry PA, Ng LFP (2020). The trinity of COVID-19: Immunity, inflammation and intervention. Nat. Rev. Immunol..

[5] Quinti I (2020). A possible role for B cells in COVID-19? Lesson from patients with agammaglobulinemia. J. Allergy Clin. Immunol..

[6] D’Onofrio V (2022). Studying the clinical, radiological, histological, microbiological, and immunological evolution during the different COVID-19 disease stages using minimal invasive autopsy. Sci. Rep..

[7] Liu PP, Blet A, Smyth D, Li H (2020). The science underlying COVID-19: Implications for the cardiovascular system. Circulation.

[8] Tang N (2020). Anticoagulant treatment is associated with decreased mortality in severe coronavirus disease 2019 patients with coagulopathy. J. Thromb. Haemost..

[9] Porfidia A, Pola R (2020). Venous thromboembolism in COVID-19 patients. J. Thromb. Haemost..

[10] Gandhi RT, Lynch JB, Del Rio C (2020). Mild or moderate Covid-19. N. Engl. J. Med..

[11] Alhazzani W (2020). Surviving sepsis campaign: Guidelines on the management of critically ill adults with coronavirus disease 2019 (COVID-19). Intensive Care Med..

[12] Xie J (2020). Association between hypoxemia and mortality in patients with COVID-19. Mayo Clin. Proc..

[13] Ahnach M, Zbiri S, Nejjari S, Ousti F, Elkettani C (2020). C-reactive protein as an early predictor of COVID-19 severity. J. Med. Biochem..

[14] Chen N (2020). Epidemiological and clinical characteristics of 99 cases of 2019 novel coronavirus pneumonia in Wuhan, China: A descriptive study. Lancet.

[15] Wu C (2020). Risk factors associated with acute respiratory distress syndrome and death in patients with coronavirus disease 2019 pneumonia in Wuhan. China JAMA Internal Med..

[16] Liu T (2020). The role of interleukin-6 in monitoring severe case of coronavirus disease 2019. EMBO Mol. Med..

[17] Panda SK, Colonna M (2019). Innate lymphoid cells in mucosal immunity. Front. Immunol..

[18] Mehta P (2020). COVID-19: Consider cytokine storm syndromes and immunosuppression. Lancet.

[19] Zhou F (2020). Clinical course and risk factors for mortality of adult inpatients with COVID-19 in Wuhan, China: A retrospective cohort study. Lancet.

[20] Vacharathit V (2021). SARS-CoV-2 neutralizing antibodies decline over one year and patients with severe COVID-19 pneumonia display a unique cytokine profile. Int. J. Infect. Dis..

[21] Faria JAQA, de Andrade C, Goes AM, Rodrigues MA, Gomes DA (2016). Effects of different ligands on epidermal growth factor receptor (EGFR) nuclear translocation. Biochem. Biophys. Res. Commun..

[22] Raab G, Klagsbrun M (1997). Heparin-binding EGF-like growth factor. Biochim. Biophys. Acta.

[23] Singh, B., Carpenter, G. & Coffey, R. J. EGF receptor ligands: Recent advances. Preprint at 10.12688/f1000research.9025.1 (2016). 10.12688/f1000research.9025.1 PMC5017282 27635238

[24] Huang C (2020). Clinical features of patients infected with 2019 novel coronavirus in Wuhan. China. Lancet.

[25] Benjamin D, Knobloch TJ, Dayton MA (1992). Human B-cell interleukin-10: B-cell lines derived from patients with acquired immunodeficiency syndrome and Burkitt’s lymphoma constitutively secrete large quantities of interleukin-10. Blood.

[26] Thompson-Snipes L (1991). Interleukin 10: A novel stimulatory factor for mast cells and their progenitors. J. Exp. Med..

[27] de Bruin S (2021). Clinical features and prognostic factors in Covid-19: A prospective cohort study. EBioMedicine.

[28] Wool GD, Miller JL (2021). The impact of COVID-19 disease on platelets and coagulation. Pathobiology.

[29] Qin C (2020). Dysregulation of immune response in patients with coronavirus 2019 (COVID-19) in Wuhan. China. Clin. Infect. Dis..

[30] Ebihara, T. *et al.* Cytokine elevation in severe COVID-19 from longitudinal proteomics analysis: Comparison with sepsis. *Front. Immunol.* **12**, (2022). 10.3389/fimmu.2021.798338 PMC8790049 35095877

[31] Ershler W, Keller E (2000). Age-associated increased interleukin-6 gene expression, late-life diseases, and frailty. Annu. Rev. Med..

[32] Tartaro DL (2022). Molecular and cellular immune features of aged patients with severe COVID-19 pneumonia. Commun. Biol..

[33] Ling, L. *et al.* Longitudinal cytokine profile in patients with mild to critical COVID-19. *Front. Immunol.* **12**, (2021). 10.3389/fimmu.2021.763292 PMC8685399 34938289

[34] Yao Y (2020). D-dimer as a biomarker for disease severity and mortality in COVID-19 patients: A case control study. J. Intensive Care.

[35] Zhang L (2020). D-dimer levels on admission to predict in-hospital mortality in patients with Covid-19. J. Thromb. Haemost..

[36] Qeadan F (2021). Prognostic values of serum ferritin and D-dimer trajectory in patients with COVID-19. Viruses.

[37] Poudel A (2021). D-dimer as a biomarker for assessment of COVID-19 prognosis: D-dimer levels on admission and its role in predicting disease outcome in hospitalized patients with COVID-19. PLoS ONE.

[38] Myhre PL (2020). Growth differentiation factor 15 provides prognostic information superior to established cardiovascular and inflammatory biomarkers in unselected patients hospitalized with COVID-19. Circulation.

[39] Lawson C, Ainsworth M, Yacoub M, Rose M (1999). Ligation of ICAM-1 on endothelial cells leads to expression of VCAM-1 via a nuclear factor-κB-independent mechanism. J. Immunol..

[40] Yin X-X, Zheng X-R, Peng W, Wu M-L, Mao X-Y (2020). Vascular endothelial growth factor (VEGF) as a vital target for brain inflammation during the COVID-19 outbreak. ACS Chem. Neurosci..

[41] Kaner RJ (2000). Lung overexpression of the vascular endothelial growth factor gene induces pulmonary edema. Am. J. Respir. Cell Mol. Biol..

[42] Marti HH, Risau W (1998). Systemic hypoxia changes the organ-specific distribution of vascular endothelial growth factor and its receptors. Proc. Natl. Acad. Sci. USA.

[43] Tamayo-Velasco Á (2021). HGF, IL-1α, and IL-27 are robust biomarkers in early severity stratification of COVID-19 patients. J. Clin. Med..

[44] Perreau M (2021). The cytokines HGF and CXCL13 predict the severity and the mortality in COVID-19 patients. Nat. Commun..

[45] Guo J (2021). Cytokine Signature Associated With Disease Severity in COVID-19. Front. Immunol..

[46] Iwamoto R, Mekada E (2000). Heparin-binding EGF-like growth factor: A juxtacrine growth factor. Cytokine Growth Factor Rev..

[47] Chobotova K (2005). Heparin-binding epidermal growth factor and its receptors mediate decidualization and potentiate survival of human endometrial stromal cells. J. Clin. Endocrinol. Metab..

[48] Young SL (2002). In vivo and in vitro evidence suggest that HB-EGF regulates endometrial expression of human decay-accelerating factor. J. Clin. Endocrinol. Metab..

[49] Asakura M (2002). Cardiac hypertrophy is inhibited by antagonism of ADAM12 processing of HB-EGF: Metalloproteinase inhibitors as a new therapy. Nat. Med..

[50] Lee K-S, Park J-H, Lim H-J, Park H-Y (2011). HB-EGF induces cardiomyocyte hypertrophy via an ERK5-MEF2A-COX2 signaling pathway. Cell Signal.

[51] Miyagawa J (1995). Localization of heparin-binding EGF-like growth factor in the smooth muscle cells and macrophages of human atherosclerotic plaques. J. Clin. Invest..

[52] Sánchez-Vizcaíno E (2010). Heparin-binding EGF-like growth factor in human serum. Association with high blood cholesterol and heart hypertrophy. Growth Factors.

[53] Tsuchida S (2018). Anti-HB-EGF antibody-mediated delivery of siRNA to atherosclerotic lesions in mice. Int. Heart J..

[54] Hsieh C-H (2017). A targetable HB-EGF–CITED4 axis controls oncogenesis in lung cancer. Oncogene.

[55] Wang L (2020). HB-EGF Activates the EGFR/HIF-1α pathway to induce proliferation of arsenic-transformed cells and tumor growth. Front. Oncol..

[56] Eapen MS (2019). Heparin-binding epidermal growth factor (HB-EGF) drives EMT in patients with COPD: Implications for disease pathogenesis and novel therapies. Lab Invest..

[57] Li Y (2021). HB-EGF-induced IL-8 secretion from airway epithelium leads to lung fibroblast proliferation and migration. BMC Pulm. Med..

[58] Pan LH, Ohtani H, Yamauchi K, Nagura H (1996). Co-expression of TNF alpha and IL-1 beta in human acute pulmonary fibrotic diseases: An immunohistochemical analysis. Pathol. Int..

[59] Kimura Y (2021). Identification of serum prognostic biomarkers of severe COVID-19 using a quantitative proteomic approach. Sci. Rep..

[60] COvid-19 Multi-omics Blood ATlas (COMBAT) Consortium. Electronic address: julian.knight@well.ox.ac.uk & COvid-19 Multi-omics Blood ATlas (COMBAT) Consortium. A blood atlas of COVID-19 defines hallmarks of disease severity and specificity. *Cell* 185, 916–938.e58 (2022). 10.1016/j.cell.2022.01.012 PMC8776501 35216673

