# Supplementary material for: Serum biomarkers associated with SARS-CoV-2 severity
Source: Sci Rep. 2022 Sep 26;12:15999. doi: 10.1038/s41598-022-20062-5 (PMC9511452; doi:10.1038/s41598-022-20062-5)
Supplement: Supplementary file 1 — Supplementary Information. [file 41598_2022_20062_MOESM1_ESM.pdf]

SUPPLEMENTARY MATERIAL

Supplementary Table 1 - List of all biological markers that we analyzed the sérum concentration levels (pg/mL) according to each MILLIPLEX Map panel. (Total=74)

**1 - Human Cytokine/Chemokine/Growth Factor Panel A Magnetic Bead Panel (Total=47)**	**2 - Human Cardiovascular Disease (CVD) Magnetic Bead Panel 2 (Total=10)**	**3 - Human Angiogenesis/Growth Factor Magnetic Bead Panel 1 (Total=17)**
sCD40L	ADAMTS13	EGF
IL-12 (p70)	D-DIMER	Angiopoietin-2
EGF	GDF-15	G-CSF
IL-13	Myoglobin	BMP-9
Eotaxin	sICAM-1	Endoglin
IL-15	MPO	Endothelin-1
FGF-2	P-SELECTIN	Leptin
IL-17A	lipocalin-2/NGAL	FGF-1
FLT-3L	sVCAM-1	Follistatin
IL-17E/IL-25	SAA	IL-8
Fractalkine	-	HGF
IL-17F	-	HB-EGF
G-CSF	-	PLGF
IL-18	-	VEGF-C
GM-CSF	-	VEGF-D
IL-22	-	FGF-2
GROα	-	VEGF-A
IL-27	-	-
IFNα2	-	-
IP-10	-	-
IFNγ	-	-
MCP-1	-	-
IL-1α	-	-
MCP-3	-	-
IL-1β	-	-
M-CSF	-	-
IL-1RA	-	-
MDC	-	-
IL-2	-	-
MIG	-	-
IL-3	-	-
MIP-1α	-	-
IL-4	-	-
MIP-1β	-	-
IL-5	-	-
PDGF-AA	-	-
IL-6	-	-

PDGF-AB/BB	-	-
IL-7	-	-
IL-8	-	-
TGFα	-	-
IL-9	-	-
TNFα	-	-
IL-10	-	-
TNFβ	-	-
IL-12 (p40)	-	-
VEGF-A	-	-
